# Identification of new genetic alterations in MLH1, MSH2 and MSH6 using IHC and HRM analysis in Lynch syndrome-suspected patients

**DOI:** 10.1186/1897-4287-10-S3-A11

**Published:** 2012-04-20

**Authors:** A Mazurek, A Fiszer-Kierzkowska, M Budryk

**Affiliations:** 1 Maria Sklodowska-Curie Memorial Cancer Center and Institute of Oncology, Gliwice, Poland; 2 Ludwik Hirszfeld Institute of Immunology and Experimental Therapy, Polish Academy of Sciences, Wrocław, Poland

## 

Mutations in DNA MMR genes, mainly MSH2 and MLH1, are the most frequent cause of HNPCC, an autosomal dominant predisposition to colorectal cancer and other malignancies. In our study we tested 46 unrelated patients with suspected HNPCC, who met Bethesda criteria. Tumors from probands (when available) were tested by immunohistochemistry for deficiencies in MLH1, PMS2, MSH2 and MSH6. DNA samples were analyzed using high-resolution melting (HRM). PCR amplicons were designed to scan complete MLH1, MSH2 and MSH6 coding sequences using HRM method. In the first stage of screening HRM analysis was performed by scanning 14 amplicons selected as potentially harboring most frequent mutations in Polish population (Kurzawski et al., 2005). Sequencing was used to confirm and characterize affected exons identified in HRM.

Three novel deleterious mutations were found in MSH2 gene, one of them being splice acceptor site mutation in exon 5, and two of them being nonsense mutations in exons 6 and 8. Tumours from patients bearing these mutations were lacking MSH2 protein. We found also missense mutation in exon 8 of MLH1, which has not been previously reported in Polish population. Tumour from this patient exhibited weak expression of MLH1 and PMS2 proteins. In 42 patients, only unspecified variants and polymorphisms have been found so far (analysis is still in progress).

In our opinion, HRM is a rapid, inexpensive and high-throughput method to prescreen for point mutation and small deletions in MMR genes. Technical aspects concerning analysis of one-replicate versus 2-replicates data will be discussed. It must be noted that HRM cannot be used alone, MLPA and array-CGH are still required for detection of large deletions and chromosome rearrangements.

